# The ubiquitin system, disease, and drug discovery

**DOI:** 10.1186/1471-2091-9-S1-S7

**Published:** 2008-10-21

**Authors:** Matthew D Petroski

**Affiliations:** 1 Rigel Pharmaceuticals, Inc., South San Francisco, CA, 94080, USA; 2 Signal Transduction Program, Burnham Institute for Medical Research, La Jolla, CA, 92037, USA

## Abstract

The ubiquitin system of protein modification has emerged as a crucial mechanism involved in the regulation of a wide array of cellular processes. As our knowledge of the pathways in this system has grown, so have the ties between the protein ubiquitin and human disease. The power of the ubiquitin system for therapeutic benefit blossomed with the approval of the proteasome inhibitor Velcade in 2003 by the FDA. Current drug discovery activities in the ubiquitin system seek to (i) expand the development of new proteasome inhibitors with distinct mechanisms of action and improved bioavailability, and (ii) validate new targets. This review summarizes our current understanding of the role of the ubiquitin system in various human diseases ranging from cancer, viral infection and neurodegenerative disorders to muscle wasting, diabetes and inflammation. I provide an introduction to the ubiquitin system, highlight some emerging relationships between the ubiquitin system and disease, and discuss current and future efforts to harness aspects of this potentially powerful system for improving human health.

Republished from Current BioData's Targeted Proteins database (TPdb; ).

## Broad overview of family

### Overview of the ubiquitin system

The ubiquitin system is a hierarchical enzymatic cascade in which a ubiquitin-activating enzyme (E1) activates the 76 amino acid protein UBIQ (ubiquitin) in an ATP-dependent manner and transfers it to the active site cysteine of ubiquitin-conjugating enzymes (E2s) [1]. Ubiquitin ligases (E3s) have a central role in the process of protein modification with UBIQ (known as 'ubiquitination' or 'ubiquitylation'); they recognize specific substrates and facilitate UBIQ transfer from the E2 onto the substrate. Although the precise number of human E3s is unknown, about 500 or more have been proposed to exist [2-5], supportive of the broad role for the ubiquitin system in regulating diverse cellular processes. Ubiquitin-like proteins (UBLs) have also been identified with varying degrees of identity to UBIQ and are conjugated onto proteins through similar enzymatic cascades as UBIQ.

Numerous deubiquitylating enzymes (DUBs) have roles in processing polyubiquitin precursor proteins and may also have regulatory roles, e.g. counteracting the ubiquitylation of a particular protein by its cognate E3 and/or proofreading synthesized UBIQ chains. There are also emerging roles for DUBs in disease (see [6]). Ubiquitin binding proteins also have diverse functions and may represent viable therapeutic targets (see [7]). In a general sense, they act as 'effector' proteins that sense a protein's modification with UBIQ and facilitate downstream signaling.

Two major classes of E3s have been identified and this classification is largely based on how they facilitate UBIQ transfer from E2 onto substrate. HECT (homologous to E6AP C-terminus) domain E3s form a catalytic UBIQ intermediate on a conserved cysteine residue prior to covalent UBIQ transfer (see [8]). The second class of E3s, which contains RING-type and structurally related ligases, facilitates the direct transfer of UBIQ from E2 onto substrate. In general, E3s facilitate covalent UBIQ transfer by properly positioning the site to be modified (i.e. a lysine residue of its recognized substrate) such that it can perform nucleophilic attack of a thioesterified UBIQ molecule either on the active site of the E2 for RING-type E3s or on the conserved cysteine of HECT domain E3s, resulting in isopeptide bond formation [9].

Lysine residues appear to be major sites of UBIQ attachment on proteins, although N-terminal and cysteine modifications have also been reported [10-17]. The type of UBIQ modification could confer distinct encoded protein fate and we are only beginning to understand how this process occurs and how it is recognized and interpreted. Mono-ubiquitylation (i.e. the attachment of a single UBIQ molecule to a single site on a protein) may be involved in histone regulation, receptor endocystosis and signaling [18-22]. UBIQ chains using a lysine residue of one UBIQ molecule attached via an isopeptide bond to the C-terminus of another UBIQ molecule add further complexity to UBIQ-encoded protein fate. Lys48-linked UBIQ chains can trigger degradation by the 26S proteasome [23-26] and Lys63-linked UBIQ chains may regulate signaling pathways [27-30] when attached to a protein. Other types of linkages (including those containing heterogeneous mixtures of linkages or branched chains) could also exist [31-33]; however their roles and physiological significance are currently unclear.

## Target validation

### Implication of the ubiquitin system in human disease

The basic functions of the UBIQ (ubiquitin) protein were first described in 1980 [34-36], yet its implication in human disease has only recently started to become appreciated. Below, I describe some relationships between the ubiquitin system and various human diseases.

### Cancer is associated with alterations in UBIQ-dependent regulation

The ubiquitin system has a widely appreciated role in regulating cellular proliferation. As expected and described in the examples below, alterations in specific pathways involving UBIQ have been associated with cancer.

#### The stability of P53 (p53) is regulated by ubiquitin ligases and a deubiquitylating enzyme (DUB)

The transcription factor P53 has a crucial role in cellular anticancer mechanisms and it has been estimated that >50% of tumors contain mutations in the P53 gene [37]. MDM2 is a major regulator of P53 function – it binds directly to P53 and targets P53 for degradation through its RING ubiquitin ligase activity [38-42]. MDM2-P53 binding, MDM2-dependent P53 degradation by the proteasome, and P53 ubiquitylation by MDM2 have been demonstrated in cell-based and *in vitro *systems by a large number of groups.

P53 regulates the stability of the interaction between MDM2 and the DUB known as UBP7 (also known as USP7, HAUSP, herpesvirus-associated ubiquitin-specific protease [43]) [44-47]. Work from the laboratory of Wei Gu demonstrated that partial reduction of UBP7 by RNAi in human cell lines (NHF-1, IMR90, U2OS and H1299 cells were tested) promotes decreased levels of both MDM2 and P53 [45]. By contrast, however, total reduction of UBP7 decreases MDM2 yet stabilizes P53. This observation suggests that the absence of UBP7 promotes MDM2 downregulation, which in turn eliminates MDM2's function as the ubiquitin ligase for P53. This mechanism requires MDM2-dependent function, as depletion of UBP7 in cells inactive for MDM2-dependent P53 turnover (i.e. HeLa cells [48]) results in P53 stabilization.

#### Cullin-RING ubiquitin ligases and the APC/C regulate cellular proliferation

Cullin-RING ubiquitin ligases (CRLs) and the anaphase-promoting complex/cyclosome (APC/C) are multi-subunit RING ubiquitin ligases that have fundamental roles in controlling the eukaryotic cell cycle (see [49]). CRLs contain a cullin protein (CUL1, CUL2, CUL3, CUL4A, CUL5, CUL7) that binds within the cullin homology domain to the RING protein RBX1 [50-52]. The distinct N-terminal regions of the cullins interact with specific classes of substrate receptors that promote the recruitment of a large number of proteins for ubiquitylation [53]. The APC/C consists of at least 11 subunits and has a mass of 1.5 mDa [54,55]. One of its subunits, APC2, contains a domain similar to cullins and associates with the RING protein APC11, suggesting that its enzymatic core is similar to CRLs [56-62].

The CUL1-based ubiquitin ligase known as SCF (SKP1-CUL1-F-box) recognizes its substrates through various receptor proteins containing the F-box motif [63]. The SKP2 F-box protein, which functions with SCF in the ubiquitylation of CDN1B (the cyclin-dependent kinase inhibitor p27) at the G_1_/S transition of the cell cycle [64-66], has garnered attention as a potential oncology target. SKP2, in conjunction with the adapter protein CKS1, recognizes phosphorylated CDN1B late in G_1_, recruiting it for ubiquitylation by SCF [67,68]. An inverse correlation between SKP2 overexpression and low CDN1B levels has been found in a variety of human tumors and transgenic mouse models, and has led to the proposal that *SKP2 *is a proto-oncogene [69-74].

The protein encoded by the tumor suppressor gene *VHL *(von Hippel-Lindau) serves as a substrate receptor for a CUL2-based ubiquitin ligase [50,75-80]. Mutations in VHL are associated with lung cancer, sporadic clear cell renal carcinomas and an autosomal dominant familial cancer known as von Hippel-Lindau disease ([81-93] and see [94]). Many of these mutations prevent VHL associating with the other subunits of its ubiquitin ligase, as judged by *in vitro *binding and co-immunoprecipitation experiments [79,80,95]. A substrate for this ubiquitin ligase is a marker of tumor hypoxia, the transcription factor HIF1A (HIF1α, hypoxia-inducible factor 1α), which stimulates angiogenesis [96]. Numerous biochemical and structural studies have determined that HIF1A binds to VHL when hydroxylated on two proline residues through the activity of prolyl hydroxylases, which results in its ubiquitylation and ultimate degradation [77,78,97-102]. Transgenic mice overexpressing a stabilized form of HIF1A that cannot be recognized by VHL develop hyper-vascularity without leakage or inflammation [103].

#### Cervical cancer is linked to HPV infection and involves downregulation of P53 and RB (Rb)

HPVs encode two oncogenic proteins known as E6 and E7, and the sexually transmitted types of HPV have a strong association with cervical cancer (see [104]). Whereas E7 may facilitate the degradation of the tumor suppressor RB through an unclear mechanism, the role of E6 in cellular transformation is more established [105]. E6 binds to a cellular protein known as UBE3A (E6AP), which is a HECT domain ubiquitin ligase [106]. This interaction promotes the recruitment of the tumor suppressor P53 to this complex, resulting in its ubiquitylation and subsequent degradation by the 26S proteasome [107,108].

#### Colorectal cancers are associated with defects in the regulation of CTNB1 (β-catenin) stability through mutations in adenomatous polyposis coli

The tumor suppressor gene *adenomatous polyposis coli *(*APC*, not to be confused with the ubiquitin ligase APC/C, anaphase-promoting complex/cyclosome, described above) is frequently mutated in colorectal cancers [109-114]. Many of these mutations truncate APC and/or alter its ability to interact with proteins, which may lead to altered regulation of cellular proliferation.

One major target subjected to regulation through APC is CTNB1, a crucial component of *Wnt *signaling and cell adhesion [115,116]. The phosphorylation of CTNB1 through APC-associated kinase activity promotes its recognition by the F-box protein β-TRCP [117,118]. Numerous cell-based and *in vitro *experiments have demonstrated that the F-box motif of β-TRCP interacts with SKP1 and assembles into an SCF complex with CUL1 [63,117-119]. Overexpression of β-TRCP containing an F-box deletion results in an increased stability of CTNB1, as demonstrated in pulse chase experiments [117,119]. Other studies have identified the RING protein SIAH1 (a *Drosophila *seven in absentia homolog) as a P53-inducible and APC-associated ubiquitin ligase that can also regulate the stability of CTNB1 [120,121].

#### Mutations in the BRCA1 ubiquitin ligase complex correlate with breast and ovarian cancer

Germline mutations in the gene encoding the RING protein BRCA1 are associated with the inherited predisposition for breast and ovarian cancer [122-124]. BRCA1 forms a heterodimer with another RING protein known as BARD1 and this complex has E3 activity *in vitro *[125-128]. These studies, utilizing bacterially expressed proteins and overexpression in mammalian cells, demonstrated that the RING motif of BRCA1 serves as the binding site for the E2 enzyme UB2D3 (UbcH5c) and that BRCA1/BARD1 together have an increased ability to conjugate UBIQ with UB2D3 *in vitro *than BRCA1 alone. Rachel Klevit's group determined that several of the cancer-predisposing mutations in BRCA1 result in defective E3 activity *in vitro *by disrupting BRCA1/BARD1 heterodimer formation or by altering the RING domain structure of BRCA1 [129-131]. Cell-based overexpression experiments demonstrated that the abundance of each protein is dependent upon the presence of its binding partner.

Phosphorylated RBBP8 (CtIP) is a reported substrate for ubiquitylation by BRCA1 [132]. Originally identified in a yeast two-hybrid screen for proteins that bind to the BRCA1 C-terminus (BRCT) domain of BRCA1 and confirmed through *in vitro *binding experiments, RBBP8 interacts with BRCA1 during G_2 _of the cell cycle [133-135]. Cell-based and *in vitro *ubiquitylation assays have demonstrated that BRCA1 ubiquitylates phosphorylated RBBP8 [132]. Rather than promoting RBBP8 degradation by the proteasome, UBIQ modification may cause RBBP8 to associate with chromatin following DNA damage and to regulate the G_2_/M transition of the cell cycle, as determined by cellular localization studies in response to DNA damage [132,136]. BRCA1 could also be associated with the DNA repair activities of the Fanconi anemia (FA) pathway (see next section), as both physical and functional interactions between BRCA1 and FA complex proteins in response to DNA damage have been described [137].

#### The Fanconi anemia pathway involves a ubiquitin ligase complex and is associated with increased cancer susceptibility

As described in the [138], studies on the rare autosomal recessive genetic disorder known as Fanconi anemia (FA) have identified a pathway crucial for the cellular response to DNA damage [139-151]. Alterations in this pathway promote increased susceptibility to cancer and have been associated with a wide variety of tumor types, even in non-FA patients [152-166]. Upon DNA damage, two proteins in this pathway are mono-ubiquitylated; FACD2 (FANCD2) and FANCI [137,141], and recruited to chromatin within nuclear foci. These nuclear foci contain other DNA repair proteins, suggesting that they are sites of DNA damage [137,147,167-169].

The role and exact molecular mechanisms underlying the regulation of FACD2 and FANCI mono-ubiquitylation are unclear; however, a protein complex (the FA core complex) contains a subunit known as FANCL that contains a RING motif and likely confers their modification [140]. Work from the D'Andrea and Dutta laboratories identified a ubiquitin-conjugating enzyme, UBE2T, that binds directly to FANCL in a yeast two-hybrid screen and through *in vitro *pull-down experiments with bacterially expressed proteins [170]. SiRNA depletion of UBE2T in U2OS cells diminished the mono-ubiquitylation of FACD2 in response to DNA damage and promoted the formation of abnormal chromosomes [170]. An siRNA screen for DUBs important for removing UBIQ from FACD2 implicated UBP1 (USP1) as an enzyme that could attenuate the role of FACD2 in DNA damage repair [171].

### Viruses exploit the ubiquitin system

Viruses utilize clever mechanisms to exploit their host to facilitate their own propagation. Modification of proteins with UBIQ during infection promotes viral replication and immune response evasion, suggesting potential anti-viral strategies.

#### HIV encodes proteins that hijack cellular cullin-RING ubiquitin ligases

The rapid evolution of viral subtypes resistant to available treatments suggests that there is still significant need for new anti-HIV therapeutics. Two HIV-encoded proteins, VIF and VPU, interact with distinct cullin-RING ubiquitin ligases to hijack their activity and promote the ubiquitylation of cellular proteins.

VIF interacts directly with a cellular cytidine deaminase, ABC3G (APOBEC3G), and facilitates its proteasome-dependent degradation [172,173]. In the absence of VIF, ABC3G is packaged into progeny virion particles, which renders them defective in replication [172,174,175]. Immunoprecipitation of hemagglutinin (HA)-tagged VIF from H9 cells (human T-cell line) infected with engineered HIV, followed by mass spectrometry, demonstrated that VIF associates with CUL5, ELOB (elongin B) and ELOC (elongin C). Western blotting was used to confirm the presence of all proteins including RBX1 [176]. *In vitro *ubiquitylation of ABC3G purified from transfected cells has been demonstrated using a reconstituted complex of these proteins from baculovirus-infected insect cells [177,178].

The CD4 cell surface receptor found on a subclass of T-cells is downregulated through the activity of VPU [179,180]. As CD4 is a co-receptor for HIV entry into cells, this downregulation could optimize viral replication by blocking further infection, allow for progeny viral particles to be efficiently released, and promote immune response evasion [179]. Co-expression of VPU and CD4 in HeLa cells results in the degradation of CD4, which can be blocked by the proteasome inhibitor MG132 [181]. Work by Margottin *et al*. originally identified the F-box protein β-TRCP from a yeast two-hybrid screen for VPU-interacting proteins [182]. After demonstrating the formation of the ternary CD4/VPU/β-TRCP complex by overexpressing these proteins in HeLa cells, the authors showed that the F-box motif of β-TRCP is necessary for CD4 degradation in these cells. It was later reported that VPU may block the ubiquitylation of IKBA (Iκbα) and CTNB1, which are phosphorylation-dependent cellular substrates of SCF^β-TRCP^, and that VPU itself may also be ubiquitylated by this complex [183-186].

#### Herpesviruses encode ubiquitin ligases and modulate cellular ubiquitin ligases

Herpesviruses often employ strategies to utilize the host cell's ubiquitin system for their own benefit. The gammaherpesvirus Kaposi's sarcoma herpesvirus (KSHV, alternatively human herpesvirus types 8, HHV8), which is associated with AIDS-related cancer (see [187]), encodes two ubiquitin ligases; K3 (MIR1) and K5 (MIR2), which downregulate a wide range of immunoreceptors (MHC class I, ICAM1, CD86, CD1D (CD1d)) from the surface of infected cells [188-193]. This mechanism could promote immune system evasion by blocking detection by cytotoxic T-lymphocytes.

The molecular details of how K3 and K5 promote immunoreceptor downregulation through the ubiquitin system are beginning to emerge. Laurent Coscoy's group recently reported the unexpected observation that transient expression of K3 in human BJAB cells stably expressing the MHC class I allele HLA.B7 can lead to downregulation of this receptor in the absence of cytoplasmic lysine residues if a cysteine residue is present [17]. Paul Lehner's laboratory used siRNA experiments to identify UBC13 and UB2D2/UB2D3 (UbcH5b/c) as the E2 enzymes important for K3-dependent downregulation of MHC class I [194]. This study suggests that K3-mediated modification of MHC class I occurs through a sequential mechanism in which Lys63-linked UBIQ chains synthesized by UBC13 are added after initial mono-ubiquitylation by UB2D2/UB2D3, thereby promoting receptor endocytosis and lysosomal targeting.

Herpesviruses also encode proteins that modulate cellular ubiquitin ligase activity, such as LMP1 (latent membrane protein 1) – encoded by EBV. LMP1 is required for EBV latency in B-cells and is sufficient to induce transformation [195,196]. Recent work from Joseph Pagano's laboratory has uncovered differential effects of LMP1 on the SIAH1 ubiquitin ligase, dependent upon cell type [197-199]. EBV-positive B-cells expressing LMP1 or cells transiently transfected to express LMP1 manifest an upregulation of CTNB1, a component of the *Wnt *signaling pathway whose increased stability has been associated with cancer (see section on *Colorectal cancers are associated with defects in the regulation of CTNB1 (β-catenin) stability through mutations in adenomatous polyposis coli*) [197]. This observation was attributed to LMP1-mediated downregulation of SIAH1, a component of a RING ubiquitin ligase complex that regulates CTNB1 stability, at the transcriptional level [120,121]. By contrast, human epithelial cells expressing LMP1 manifest increased SIAH1 protein levels and a resulting decrease in the SIAH1 substrates prolyl hydroxylases 1 and 3 (PHD1, PHD3 [also known as EGLN2 and EGLN3]) [198,200]. These decreases promote the stability of the transcription factor HIF1A as it cannot be hydroxylated, an event required for its association with the VHL-containing ubiquitin ligase (see section on *Cullin-RING ubiquitin ligases and the APC/C regulate cellular proliferation*).

### Neurodegenerative diseases often have associated impairment of the ubiquitin system

The formation of protein aggregates containing UBIQ has long been associated with neurodegenerative diseases such as Parkinson's, Alzheimer's, Huntington's, and others. For example, polyglutamine repeat expansion in proteins associated with Huntington's disease and the spinocerebellar ataxias could promote the formation of protein aggregates that are resistant to degradation by the proteasome and also impair proteasome function (see [201]). Similarly in Alzheimer's disease, the formation of neurofibrillary tangles and plaques associated with amyloid-β protein aggregation and/or ubiquitylated TAU (tau) accumulation could impair proteasome function (see [202]).

Another example is found with autosomal-recessive juvenile Parkinson's disease, in which mutations in the ubiquitin ligase PRKN2 (parkin) manifest as defects in its ligase activity *in vitro *[203-205], suggesting that accumulation of its substrates could contribute to disease development.

### Metabolic diseases such as diabetes could have associated defects in aspects of the ubiquitin system

The exact relationships between the ubiquitin system and metabolic processes are only beginning to be understood (see [206]). Insulin resistance, associated with diabetes and obesity, manifests as defects in sensing and signaling mechanisms. The ubiquitin system has been associated with insulin signaling through regulating the stability of insulin receptor substrate (IRS) proteins.

IRS proteins serve as adapter molecules, functioning between receptor tyrosine kinases and downstream signaling molecules. IRS2 in particular has a crucial function in controlling the growth and survival of pancreatic β-cells – the body's source of insulin. *Irs2 *knockout mice are diabetic and exhibit dramatic reduction in β-cell mass, and decreasing IRS2 expression via siRNA in β-cells promotes apoptosis and decreased cell survival [207]. Thus, signaling through IRS2 has a crucial function in regulating the body's response to changes in glucose.

IRS2 function is regulated by phosphorylation and UBIQ-mediated degradation by the proteasome [208,209]. In pancreatic β-cells, activation of the kinase FRAP (mTOR), as demonstrated by adenoviral delivery of constitutively active FRAP to rat INS-1 cells, promotes IRS2 phosphorylation and degradation by the proteasome [210]. IRS2 interacts with suppressors of cytokine signaling (SOCS) proteins SOCS1 and SOCS3 in human HEK293 cells, mouse 3T3-L1 adipocytes, and mouse hepatocytes [210]. These proteins contain a 'SOCS box' motif, which promotes their interaction with ELOC, a component of a cullin-based ubiquitin ligase, which in turn recruits IRS2 for ubiquitylation and targets it for degradation by the proteasome [211]. Future studies aimed at understanding how IRS2 abundance relates to mechanisms of glucose sensing may lead to novel approaches for combating the growing epidemic of diabetes.

### Muscle wasting disorders have increases in ubiquitin system function

Decreases in skeletal muscle mass associated with aging, cancer, disuse and other physiological circumstances occur through proteolytic mechanisms involving calpain proteases and UBIQ-dependent protein degradation (see [212]).

Experimental evidence suggests that numerous genes of the ubiquitin system are upregulated during muscle atrophy, including those encoding a muscle-specific ubiquitin ligase (TRI63, also known as MuRF1) and a potential substrate receptor for SCF (the F-box protein FBX32, also known as MAFbx and Atrogin-1) [213]. Knockout studies in mice performed by Regeneron for each of these genes support their crucial roles in promoting muscle atrophy [213]. Whereas TRI63 deletion results in 36% sparing of muscle mass loss after denervation of the right hindlimb muscle of mice, FBX32 deletion allows for 56% sparing under similar experimental conditions when compared with controls.

Potential substrates for these ubiquitin ligases are only beginning to be identified. TRI63 may target TITIN (titin), a protein implicated in myofibril organization for degradation [214]. The binding between TRI63 and the C-terminal region of TITIN was originally identified by yeast two-hybrid studies [215]. FBX32, as a substrate receptor for SCF, has been proposed to facilitate the ubiquitylation of the calmodulin-dependent phosphatase calcineurin A and MyoD, a transcription factor involved in myogenic differentiation [216,217]. Cam Patterson's group identified calcineurin A through a yeast two-hybrid screen using FBX32 as bait and validated this interaction through *in vitro *and cell-based experiments and overexpression studies in mice. Using MyoD as bait in a yeast two-hybrid screen, FBX32 was identified as an interacting protein, and a variety of cell-based and *in vitro *experiments demonstrated binding, ubiquitylation and turnover dependent upon this interaction [217].

### UBIQ is involved in NFκB activation to regulate inflammation and innate immunity

Numerous distinct pathways can promote the activation of NFκB in response to distinct stimuli (such inflammatory cytokines, DNA damaging agents and microbes) and UBIQ has diverse and complex roles in this process. Conventional roles for UBIQ, such as targeting the NFκB inhibitor IKBA for degradation by the proteasome [118,218-220] and the proteasome-dependent processing of NFκB precursor proteins [221,222], are mixed with non-proteolytic functions in regulating specific signaling pathways [28,30]. Also, these signaling mechanisms can be attenuated by specifically associated DUBs [223]. This complexity provides distinct levels of regulation of NFκB activation that could allow for the modulation of this process associated with a wide range of diseases. NFκB activation through TNR11 (receptor activator of NFκB, RANK), for example, has an important role in bone homeostasis and is associated with diseases such as osteoporosis, rheumatoid arthritis and Paget's disease of bone (see [224]).

The importance of regulating NFκB signaling through distinct pathways is highlighted by the tumors associated with the genetic disorder cylindromatosis [225]. Afflicted individuals contain mutations in a DUB known as CYLD. This enzyme functions downstream of the tumor necrosis factor α receptor and is involved in attenuating NFκB activation by deubiquitylating the signaling molecules TRAF2, TRAF6 and TRAF7 [226-228]. The regulation of TRAF2 signaling by CYLD was identified by screening an RNAi library targeting DUBs for ones that attenuate NFκB activation [227] and through a two-hybrid screen for proteins that interact with the regulatory subunit of the IκB kinase complex that could also bind to TRAF2 [228]. Later work studying signaling through the toll-like receptor 2 (TLR2) performed cell-based experiments to demonstrate that CYLD binds to TRAF6 and TRAF7 and that depletion of CYLD increases the ability of transfected TRAF6 or TRAF7 to activate an NFκB-dependent reporter gene [226].

### UBIQ-dependent ion channel stability has implications in cardiovascular diseases

Alterations in ion channel stability have been associated with cystic fibrosis and Liddle's syndrome. Cystic fibrosis, one of the most common genetic diseases, is characterized by a wide array of recessive mutations in CFTR (cystic fibrosis transmembrane conductance regulator), a Cl^- ^ion channel protein (see [229]). These mutations promote CFTR misfolding and subsequent clearance through protein quality control pathways of the ubiquitin system. Exactly how CFTR downregulation promotes lung disease is unclear. By contrast, Liddle's syndrome is associated with increased stability of an epithelial Na^+ ^channel (ENaC, see [230]). This autosomal dominant disorder is characterized by mutations in ENaC that block its recognition by the HECT ligase NEDD4 (see [8]), which promotes ENaC accumulation at the cell surface. Alterations of proper ionic balance in the kidney through increases in ENaC may increase blood volume and blood pressure, promoting cardiovascular disease [231,232].

## Lead discovery

### Current drug discovery activities focused on the ubiquitin system

The 26S proteasome is the only validated therapeutic target of the ubiquitin system, with a single commercially available drug known as Velcade. Current drug discovery activities related to the ubiquitin system focus on three major areas: (i) expanding indications of proteasome inhibition in therapy, (ii) developing proteasome inhibitors targeting different activities of the proteasome and with improved bio-availability, and (iii) validating the potential of other targets of the ubiquitin system.

#### Proteasome inhibition is a treatment for cancer

Changes in proteasome function have been implicated in the development of various diseases (see [233]). In 2003, a small molecule proteasome inhibitor known as Velcade (bortezomib by Millennium Pharmaceuticals) was approved by the FDA for the treatment of relapsed or refractory multiple myeloma (see [234]). Velcade is a boronic acid derivative (chemical name [(1R)-3-methyl-1-[[(2S)-1-oxo-3-phenyl-2-[(pyrazinylcarbonyl) amino]propyl]amino]butyl] boronic acid) that is a reversible inhibitor of the chymotrypsin-like activity of the 26S proteasome [235].

Efforts are underway by several other companies to develop inhibitors that target distinct activities of the proteasome. One such company is Proteolix, which has developed PR-171, a synthetic analog of epoxomycin that irreversibly inhibits the chymotryptic site of the proteasome. Treatment of xenograft models over the course of two days demonstrated that PR-171 has a stronger anti-tumor effect than Velcade [236]. Phase I trials are currently underway for PR-171 to evaluate its role in treating multiple myelomas and non-Hodgkin's lymphoma. Another proteasome-targeting drug that is currently in Phase I clinical trials is salinosporamide A (NPI-0052), developed by Nereus Pharmaceuticals. Pre-clinical studies have demonstrated that NPI-0052 achieves a higher and more sustained level of proteasome inhibition when compared with Velcade [237]. It is also well tolerated and improves the response of multi-drug treatment of a colon cancer xenograft model [237]. These two drugs highlight the therapeutic potential of proteasome inhibition when treating various cancerous states. Indeed, Cytomics Systems, Eisai, Novartis AG, Bristol-Myers Squibb, Cell Therapeutics, Cephalon and Ergon Pharmaceuticals are all developing proteasome inhibitors for this purpose.

#### Small molecules can promote P53 (p53) stability

As described in the section *Cancer is associated with alterations in UBIQ-dependent regulation*, MDM2 is a RING ubiquitin ligase that has a crucial role in regulating P53 stability. The development of inhibitors that disrupt this interaction will play a major role in the regulation of cell cycle progression and potentially the treatment of cancer. One group of small molecules currently in development is the nutlins, by Roche. These represent the first small molecules that can interfere with the ability of MDM2 to mediate P53 ubiquitylation [238]. Nutlins bind to the pocket domain of P53, and inhibit xenograft tumor growth *in vivo *with no obvious toxicity. They activate the P53 pathway at a range of 1–3 μM, stimulating cell cycle arrest and apoptosis in tumor cells. By contrast, treatment of untransformed cells results in cell cycle arrest, but no apoptosis [239].

RITA [2,5-bis(5-hydroxymethyl-2-thienyl)furan (NSC652287)], isolated from a screen by the National Cancer Institute (NCI) [240,241], is another compound that has been shown to regulate the interaction between MDM2 and P53. RITA also induces apoptosis in human tumor cells, but has little effect on normal cells. It has been proposed to bind the N-terminus of P53; thereby causing a conformational change that prevents MDM2 binding. However, the sensitivity of human fibroblasts to RITA is varied and depends on the expression level of oncogenes such as *MYC *(*C-MYC*) [242]. RITA, as with nutlins, has a significant antitumor effect on mice carrying human tumor xenografts, without apparent toxicity [243], and both are in pre-clinical development. The therapeutic potential of these and other molecules targeting this important regulatory mechanism are currently being explored. Nevertheless, they represent an important class of molecules, which block substrate recognition by a ubiquitin ligase through interfering with a protein-protein interaction interface.

#### Inhibition of E1 may be a viable therapeutic target

If inhibiting the proteasome has therapeutic utility, then perhaps targeting UBIQ (ubiquitin) activation by E1 might also be beneficial. Both Millennium Pharmaceuticals and Rigel Pharmaceuticals have filed patents disclosing their discovery of E1 inhibitors (Millennium patent: WO2006084281 [244], Rigel patent: WO2005037845 [245], see [246]). These small molecule inhibitors of UBIQ activation may also have potential as cancer therapeutics.

#### Ubiquitin ligases and deubiquitylating enzymes are emerging therapeutic targets

Proteasome inhibition or E1 inhibition appear to be global approaches to controlling protein activities regulated by UBIQ. As a result, efforts are ongoing to selectively target enzymes involved in specific ubiquitylation pathways. Ubiquitin ligases and deubiquitylating enzymes (DUBs) have gained the most attention due to their direct roles in regulating their recognized protein's stability. However, the complexity of protein ubiquitylation coupled with the absence of catalytic pockets for small molecule binding has made targeting ubiquitin ligases challenging. Nevertheless, Rigel Pharmaceuticals is currently characterizing ubiquitin ligases as potential therapeutic targets. The company has a broad, on-going program to explore the role of specific ubiquitin ligases in oncology, inflammation, virology and metabolism – with Merck and Daiichi as collaborators. Other companies such as Celgene, Amgen and Genentech also have programs exploring ubiquitin ligases for clinical indications. Thus, the flurry of activity surrounding ubiquitin ligases supports their potential therapeutic importance for developing new treatments for a wide variety of conditions.

In contrast to ubiquitin ligases, DUBs have a more simple mechanism of action and a catalytic pocket for targeted binding of small molecules. Hybrigenics recently disclosed the discovery of several small molecules that can inhibit UBP7 (USP7) (HBX 41108) and UBP8 (USP8) (HBX 90397, HBX 90659), which may have oncology applications.

## New frontiers in drug discovery for the ubiquitin system

Our understanding of the complexities of the ubiquitin system and its involvement in aspects of physiology is still developing and (perhaps justifiably) so are efforts to discover drugs targeting its enzymes. Here, I summarize some of the current challenges and opportunities that could allow for the potential of the enzymes of the ubiquitin system to be realized as therapeutic targets.

### How can ubiquitin ligase activity be inhibited?

Ubiquitin ligases may represent the best target class of the ubiquitin system due to their intrinsic specificity for particular protein substrates. The vast majority of ubiquitin ligases are RING-type. These appear to function primarily as scaffolds to position the substrate to be modified in close juxtaposition with the ubiquitin-conjugating enzyme to promote covalent UBIQ (ubiquitin) transfer [247]. Thus, means to modulate ubiquitin ligase activity may necessarily focus on targeting protein-protein interfaces. Whereas the MDM2-P53 (p53) interface can be disrupted by small molecules, is this possible for other E3-substrate interactions? Do different RING motifs (the binding site for the ubiquitin-conjugating enzyme) look different enough to be considered as targets? If so, how can they be targeted? HECT (homologous to E6AP C-terminus) domain ligases, by contrast, have a catalytic function and may undergo conformational changes [248], suggesting that, at least superficially, they could be more amenable.

### Can we harness ubiquitin ligases to artificially target proteins for ubiquitylation?

Increased protein stability correlates with several disease phenotypes. For some of these cases, defects in upstream signaling results in impaired substrate targeting to ubiquitin ligases. In other cases, the ubiquitin ligase itself has functional defects. These observations suggest that artificially recruiting a protein to a ubiquitin ligase could be an approach to restoring proper protein homeostasis. Indeed, there are several reports supporting the potential of this approach [249-251]. One such strategy has involved the development of a chimeric protein or 'ProTaC' (proteolysis targeting chimeric molecule), which targets the protein to the SCF^β-TRCP ^ubiquitin ligase. SCF^β-TRCP ^then targets the protein for ubiquitylation, followed by proteasomal degradation. ProTaC consists of a SCF^β-TRCP^-binding IKBA (IκBα) phosphopeptide linked to a domain that binds the targeted protein [252]. Further development of this technology could have the potential to realize a powerful and specific tool for the treatment of diseases such as cancer.

### Are ubiquitin-conjugating enzymes good therapeutic targets?

Whereas there are considerably fewer ubiquitin-conjugating enzymes than ubiquitin ligases, they may function in specific ubiquitylation pathways. As they represent an essential part of the ubiquitylation process, conferred through their enzymatic activity, they may be reasonable targets. However, these enzymes have a very highly conserved enzymatic core and do not possess defined catalytic pockets [253].

### What other approaches should be considered?

Recent work identified small molecules known as ubistatins that bind to Lys48-linked UBIQ chains *in vitro *and block UBIQ-dependent protein degradation by the proteasome [254]. Randy King's group at Harvard identified ubistatins in a chemical genetic screen for small molecules that stabilize Cyclin B in *Xenopus *extracts, and they were subsequently shown to inhibit the degradation of the yeast cyclin-dependent kinase inhibitor SIC1 (Sic1) in a reconstituted *in vitro *system. NMR and *in vitro *binding studies determined that these molecules bind specifically to Lys48-linked UBIQ chains. Whilst these molecules are not cell permeable and have unclear therapeutic potential, they do represent a new approach for blocking protein degradation.

Another direction could be to target mechanisms that regulate ubiquitin ligase assembly. The SCF ubiquitin ligase, for example, is comprised of multiple subunits in which substrate recognition is conferred by a variable receptor subunit (F-box proteins) [53]. The assembly of specific SCF complexes appears to be regulated at multiple levels ranging from receptor abundance to post-translational modification of its enzymatic core. As our knowledge of the intricacies of the ubiquitin system continues to grow, it is likely that we will uncover new regulatory mechanisms associated with protein ubiquitylation.

## List of abbreviations

APC: adenomatous polyposis coli; APC/C: anaphase promoting complex/cyclosome; BRCT: BRCA1 C-terminus; CFTR: cystic fibrosis transmembrane conductance regulator; CRLs: Cullin-RING ligases; DUB: deubiquitylating enzyme; E1: ubiquitin-activating enzyme; E2: ubiquitin-conjugating enzyme; E3: ubiquitin ligase; ENaC: epithelial Na^+ ^channel; FA: Fanconi anemia; HECT: homologous to E6AP C-terminus; HHV8: human herpesvirus type 8; HIF1α: hypoxia-inducible factor 1α; IRS: insulin receptor substrate; KSHV: Kaposi's sarcoma herpesvirus; ProTaC: proteolysis targeting chimeric molecule; RANK: receptor activator of NFκB; SCF: SKP1-CUL1-F-box ubiquitin ligase; UBLs: ubiquitin-like proteins.

## Competing interests

The author was employed by a pharmaceutical company with an interest in the ubiquitin system during the writing of this review.

## Publication history

Republished from Current BioData's Targeted Proteins database (TPdb; ).
